# Hazard rates of recurrence for gastric cancer after curative resection: implications for postoperative surveillance

**DOI:** 10.1007/s10120-024-01576-5

**Published:** 2024-12-26

**Authors:** Kyohei Kanematsu, Yuta Nakayama, Mie Tanabe, Junya Morita, Shinsuke Nagasawa, Takanobu Yamada, Takashi Ogata, Takashi Oshima

**Affiliations:** Department of Gastrointestinal Surgery, Kanagawa Cancer Center, 2-3-2 Nakao, Asahi Ward, Yokohama, Kanagawa 241-8515 Japan

**Keywords:** Gastric cancer, Recurrence, Hazard function, Surveillance

## Abstract

**Background:**

Identifying the most effective postoperative surveillance interval in patients with gastric cancer (GC) remains challenging. To elucidate a logical and effective surveillance schedule, we analyzed GC recurrence risk trends after gastrectomy using the hazard function.

**Methods:**

We retrospectively reviewed the medical records of 2503 patients who underwent curative GC resection between 2000 and 2018. We examined recurrence risk over time and the influence of clinicopathological variables on it.

**Results:**

Overall, GC recurred in 291 patients (11.6%) over a median of 64.6 months. Recurrence risk was highest at approximately 11-months postoperatively (hazard rate [HR]: 0.0045), decreasing to half the peak at approximately 39-months postoperatively. Patients with Stage I GC maintained a low risk. In Stage II patients, the risk peaked at 16-months postoperatively (HR: 0.006) and gradually declined thereafter. Stage III patients had the highest risk at 11 months postoperatively (HR: 0.019), plateauing at 40 months.

**Conclusions:**

We demonstrated significant cancer stage-dependent differences in postsurgical GC recurrence risk by using the hazard function. Reductions in surveillance intensity might be acceptable according to the individual risk of recurrence.

**Supplementary Information:**

The online version contains supplementary material available at 10.1007/s10120-024-01576-5.

## Introduction

Gastric cancer (GC) is the fifth most common cancer worldwide that resulted in approximately 782,700 deaths in 2018 [1]. Recent advances in surgery and perioperative chemotherapy have improved the 3-year survival rates of patients with Stage II and III GC to over 80% [2, 3].

Regular postoperative monitoring is essential for early detection of recurrence to improve patient outcomes. However, surveillance entails costs and time burdens, impacting both patients and healthcare staff. Therefore, accurate risk analysis of tumor recurrence is crucial to avoid unnecessary tests. Nevertheless, consensus on the optimal follow-up strategy remains elusive, with guidelines offering differing recommendations. For instance, while the National Comprehensive Cancer Network (NCCN) suggests biannual computed tomography (CT) scans for 2 years after surgery for patients with Stage II/III GC [4], the National Institute for Health and Care Excellence (NICE) guidelines do not recommend routine surveillance solely for recurrence detection [5].

Traditional statistical methods, such as Kaplan–Meier analysis, provide information on the cumulative incidence over time but offer limited insights into specific recurrence risks at any given point [6]. Thus, some studies have utilized the hazard functions to estimate recurrence risk continuously, focusing on patients still at risk at each time point and providing a more nuanced understanding of recurrence risk [6–8]. This approach has been proven to be beneficial for other cancer types and has provided a more subtle understanding of the risk of recurrence.

Therefore, in this study, we aimed to apply this method to patients with GC and examined changes in the hazard rates (HRs) of recurrent events over time to elucidate logical and effective surveillance schedules for patients with GC at each stage.

## Methods

### Patients

This retrospective cohort study was conducted at the Kanagawa Cancer Center Hospital, Japan. It included patients who underwent gastric resection for Stage I–III GC between January 2000 and December 2018. Baseline information, including demographic variables, pathological details, treatment specifics, postoperative conditions, and recurrence status, was extracted from a prospectively maintained database. Cancer staging was performed according to the Union for International Cancer Control tumor-node-metastasis (TNM) classification (7th edition) [9]. The extent of lymphadenectomy was classified as standardized ‘limited’ lymphadenectomy (D1) or standardized ‘extended’ lymphadenectomy (D2) in accordance with the Japanese Gastric Cancer Treatment Guidelines 2010 (ver. 3) [10]. This study was approved by the Institutional Review Board (IRB) of the Kanagawa Cancer Center Hospital (IRB code: 2023–142).

### Postoperative follow-up and definition of recurrence

Following surgery, patients generally undergo regular follow-up according to the Japanese Gastric Cancer Association (JGCA) guidelines [10]. For the first 3 years postoperatively, patients underwent physical examinations and blood tests, including serum tumor marker measurements every 3 months, followed by biannual physical examinations, up to the fifth year. Chest, abdominal, and pelvic CT scans with intravenous contrast were performed every 6–12 months for 5 years postoperatively. Upper endoscopic surveillance was performed during the first and third years postoperatively.

The date of recurrence was defined as the date on which abnormal findings were noted on the initial surveillance examination or the date of symptomatic presentation, further confirmed by additional examinations, leading to a diagnosis of recurrence. Recurrence was diagnosed based on the appearance of new lesions on CT or positron emission tomography, or histological findings from biopsies. Recurrent cases were reviewed by at least two individuals, including one radiologist and one surgeon. The recurrence status was censored at the final visit date. Metachronous remnant gastric cancer detected during follow-up period was not considered a recurrence.

### Statistical analysis

Recurrence-free survival (RFS) was defined as the time from the primary surgery to the date of the first recurrence or death from any cause until December 2022. Continuous estimation of the recurrence HRs was performed using the hazard functions of RFS, defining recurrence or death from any cause as an event. The HRs in this study were measured monthly. Time-varying HRs were estimated using the non-parametric kernel smoothing method proposed by Muller and Wang [11]. All statistical analyses were conducted by R software version 4.2.2 (https://www.r-project.org/).

## Results

### Patient and clinicopathological characteristics

Of 3,365 patients who underwent surgery for GC at our facility between 2000 and 2018, 2,503 were included. We excluded 146, 151, 271, and 91 patients with non-adenocarcinoma histology, R2 resection, surgery after chemotherapy, and palliative surgery, respectively. Table 1 shows the clinical and pathological characteristics of the patients. Follow-up was conducted until December 2023. The median follow-up period for patients who survived without recurrence by the end of the study was 64.6 months (range, 0–248 months) (Supplementary Fig. 1). Of the 826 patients with Stage II/III GC, 434 (52.5%) received adjuvant chemotherapy. During the study period, 291 patients (11.6%) experienced recurrence, corresponding to 2.4% (41/1677 cases), 16.9% (71/420 cases), and 44.1% (179/406 cases) of patients with Stage I, II, and III GC, respectively.

**Table 1:** Patient Characteristics (n = 2503)

Characteristic	Number (%)
Age, years	
Median	64
Range	24–90
Sex	
Male	1688 (67.4)
Female	815 (32.6)
Tumor location	
Upper	571 (22.8)
Middle	1143 (45.7)
Lower	789 (31.5)
Histologic grade	
Well to moderate	1151 (46.0)
Poor	1306 (52.2)
Unspecified	46 (1.8)
Adjuvant therapy in Stage II/III	
Yes	434 (52.5)
No	392 (47.5)
Type of lymph-node dissection	
D1	1210 (48.3)
D2	1195 (47.7)
Others	97 (3.9)
Cancer stage, TNM classification	
I	1677 (67.0)
II	420 (16.8)
III	406 (16.2)
Type of gastrectomy	
Total	760 (30.2)
Distal	1633 (65.2)
Proximal	43 (1.7)
Other	67 (2.7)
Number of lymph node examined	
Median	45
Range	0–147

D1, ‘limited’ lymphadenectomy; D2, standardized ‘extended’ lymphadenectomy; TNM, tumor-node-metastasis

### Overall RFS and HRs for recurrence

**Fig. 1:**
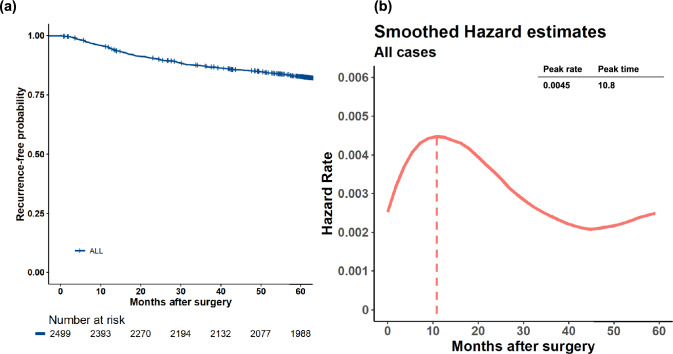
Kaplan–Meier plot (with 95% confidence intervals) of the time to recurrence (**a**) and smoothed hazard functions for gastric cancer recurrence in all patients (**b**)

Figure 1a depicts the conventional Kaplan–Meier estimates (with 95% confidence intervals) of RFS for the 2,502 patients enrolled in this study. The total number of events was 559 (RFS), including 268 deaths and 291 recurrences. When plotting HRs over time for survival duration, the risk of recurrence peaked sharply at 10.8 months post-treatment (HR 0.0045) and then decreased to less than half the peak value after 30 months (Fig. 1b).

### RFS and HRs for recurrence stratified by TNM stage

**Fig. 2:**
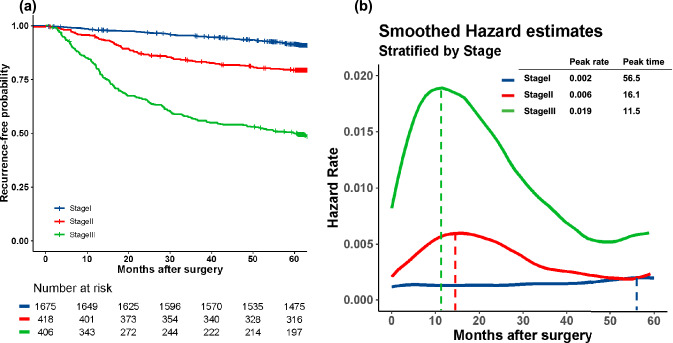
Kaplan–Meier plot of the time to recurrence in all patients (**a**) and smoothed hazard functions for gastric cancer recurrence in all patients (**b**)

Patients were stratified into groups based on the TNM stage. Figure 2a shows the RFS curves for patients stratified according to the TNM stage. The 5-year RFS rates were 91.3% (n = 1,474), 79.3% (n = 319), and 50.0% (n = 198) for Stages I, II, and III, respectively. After stratification by the TNM stage, the HRs were plotted against time (Fig. 2b) to visualize the dynamics of recurrence risk by the TNM stage. The HRs for Stage I were consistently low (< 0.002), with no apparent peaks in the curve. For Stage II GC, the peak HR was three times higher than that for Stage I GC, after which the curve gradually declined. The HR for Stage III GC increased steeply compared with Stage II GC, peaking at 11.5 months (peak HR 0.019), and remained consistently higher than that of Stage II GC throughout the surveillance period. As the stage advanced, the peak HR increased and the peak time decreased.

### HRs for recurrence stratified by lymph node dissection according to TNM stage

**Fig. 3:**
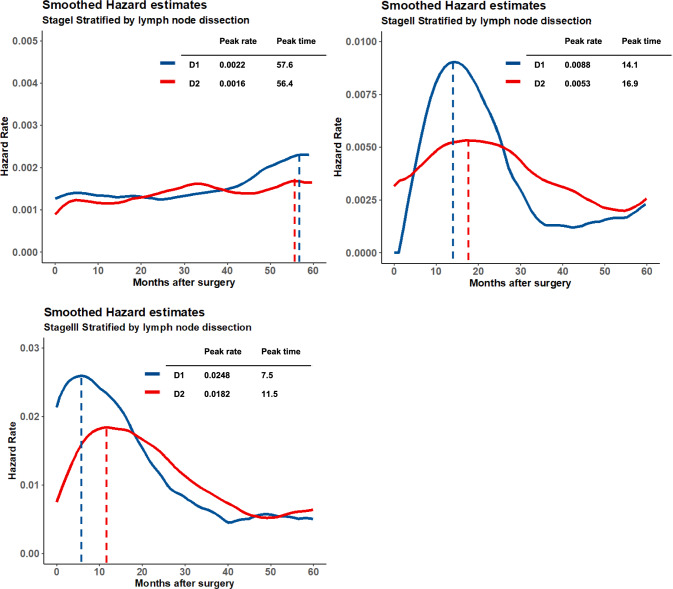
Smoothed hazard estimates for gastric cancer recurrence stratified by TNM stage and the extent of lymph node dissection

Figure 3 shows the HRs for recurrence according to the extent of lymph node dissection in patients with different TNM stages. For all stages, the peak HR for recurrence was significantly higher in the D1 group than in the D2 group. For Stages II and III, the peak time in the D1 group was shorter than that in the D2 group. In Stage I, the HR for the D1 group gradually increased from 40 months postoperatively, leading to a divergence between the D1 and D2 groups.

### Impact of surgical type, adjuvant chemotherapy with S-1 and recurrence pattern on HR for recurrence

**Fig. 4:**
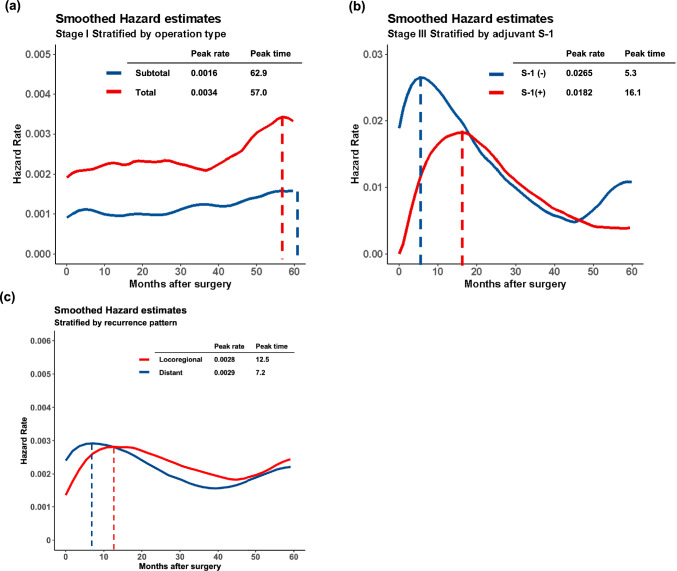
Smoothed hazard estimates for gastric cancer recurrence stratified by operation type among patients with Stage I gastric cancer (**a**). Smoothed hazard estimates for recurrence stratified by the presence or absence of adjuvant chemotherapy (S-1) in Stage III gastric cancer (**b**). Smoothed hazard estimates for recurrence stratified by recurrence pattern (Locoregional or Distant recurrence) (**c**)

Furthermore, patients were grouped based on the surgical type (total gastrectomy or subtotal gastrectomy) and postoperative adjuvant chemotherapy. Among patients with Stage I GC, those who underwent total gastrectomy had approximately twice the HR for recurrence compared with those with gastric preservation (Fig. 4a).

Further analysis was performed based on the presence of adjuvant chemotherapy with S-1 to visualize the effect of adjuvant chemotherapy on changes in the HRs. Figure 4b shows the HRs for recurrence in the Stage III cohorts of patients who received adjuvant chemotherapy with S-1 and those who did not. The HR for the non-adjuvant chemotherapy group peaked at 5.3 months (peak HR 0.0265), whereas that for the adjuvant chemotherapy group peaked at 16.1 months (peak HR 0.0182). After reaching their respective peaks, the two curves overlapped for some time, but after 45 months, the HR for the non-adjuvant group increased.

To evaluate the impact of the recurrence pattern on the hazard curves for recurrence, we categorized recurrences as locoregional (gastric bed, regional lymph nodes, peritoneal carcinomatosis, para-aortic.

or hepatic hilar lymph nodes) and distant (liver, bone, or other hematogenous metastases). Locoregional and distant recurrence occurred in 162 and 129 patients, respectively. As shown in Fig. 4c, the peak time for distant metastasis is five months earlier than that for locoregional metastasis (locoregional; peak time: 18.0 months, peak HR: 0.0035, distant; peak time: 12.9 months, peak HR: 0.0038).

## Discussion

In this study, we aimed to determine an appropriate surveillance program after gastrectomy for GC using the hazard function and showed that the risk of GC recurrence after curative surgery varies significantly according to the TNM stage and other clinicopathological factors, with a higher and earlier peak recurrence risk as the disease stage progresses. Our findings may simply confirm the evident fact that recurrence occurs earlier and more frequently as GC progresses by using the new statistical method of the hazard function. However, our results have important implications for rational post-gastrectomy surveillance programs. To the best of our knowledge, this analysis using the hazard function provides evidence regarding the optimal intensity and duration of surveillance for curatively resected GC that has not been available to date.

Current NCCN guidelines recommend frequent follow-up with medical history taking and physical examination for the first 1–2 years in patients who have undergone surgery for GC [4]. Surveillance modalities, such as CT scans, clinical tests, and upper endoscopies, are recommended only if clinically indicated for Stage I GC. For Stage II/III GC, CT scans are recommended every 6 months for the first 2 years and annually thereafter for up to 5 years. The NICE guidelines do not recommend routine surveillance, including radiological surveillance aimed only at detecting recurrence [5]. Descriptions of post-gastrectomy surveillance in other guidelines [12] are somewhat ambiguous. These recommendations in various guidelines are based on retrospectively analyzed literature and not on high-level evidence from a proper analysis of the risk of recurrence.

In our study, the HR for patients with Stage I GC remained consistently low throughout the surveillance period, suggesting that intensive surveillance may be unnecessary for patients with Stage I GC, starting from the first year postoperatively, which is consistent with the NCCN guidelines [4]. Furthermore, while the overall recurrence risk for patients with Stage II GC was lower than that for patients with Stage III GC, the peak HR was three times higher than that for Stage I. Subgroup analysis showed that, regardless of adjuvant chemotherapy or D2 lymph node dissection, the recurrence risk halved beyond 2-years postoperatively, suggesting that intensive surveillance is needed for the first 2 years after surgery for Stage II GC. In contrast, for patients with Stage III GC, the HR for recurrence halved after 3 years, suggesting the need for continued intensive surveillance for at least 3-years postoperatively.

High local recurrence suppression rates with D2 lymph node dissection were reported in a 15-year follow-up Dutch clinical trial in 2010, and D2 lymph node dissection is the standard surgical approach for patients with curable GC [3]. In the subgroup analysis, patients who underwent D1 lymph node dissection showed a significantly higher peak recurrence risk than did those who underwent D2 lymph node dissection at any stage. In our study, while the peak recurrence in the D1 group occurred 3 months earlier in Stage II and 4 months earlier in Stage III than in the D2 group, the time until the recurrence risk halved was almost the same for all cohorts, suggesting no need to change surveillance intervals based on D1 or D2 lymph node dissection, for all stages of GC.

During the study period, adjuvant chemotherapy with S-1, an oral fluoropyrimidine compound, was the standard treatment for Stage II/III GC in Japan [2]. In the Stage III cohort, the peak recurrence in patients who underwent adjuvant chemotherapy occurred 10 months later than in those who did not. However, despite the different timings of recurrence peaks, HR plots for both groups almost overlapped at approximately 20 months postoperatively. Thirty months postoperatively, the HRs for recurrence in both groups were less than half of the peak value. Therefore, intensive surveillance for 3 years postoperatively seems sufficient, regardless of adjuvant chemotherapy use. However, the resurgence of the recurrence risk at 5 years postoperatively in patients with Stage III GC who did not undergo adjuvant chemotherapy cannot be ignored. For this cohort, some forms of surveillance beyond 5 years should be considered.　Considering the recurrence pattern (Fig. 4c), the result that less symptomatic distant recurrences occur earlier than locoregional recurrences confirms the importance of intensive follow-up in the early postoperative period with imaging study.

This study used the hazard function of RFS to evaluate the trend of risk of recurrence, The effects of deaths from other diseases must be considered, and caution must be exercised when interpreting the results especially in the latter period of follow-up times. As shown in Fig. 1b, the HRs after 45 months rise again. This reflects the proportion of deaths from other diseases increases compared with the number of recurrence events (Supplementary Fig. 2). Moreover, given the univariate aspect of this analysis, some biases due to each background factor must be considered when comparing between groups. In the subgroup analysis comparing D1 and D2 in the Stage I cohort (Fig. 3a), we observed a trend of increased HRs in the D1 group after 40 months postoperatively. This trend may be an effect of death due to other diseases, and the increase of them in Stage I with the D1 cohort (Supplementary Fig. 3) may be attributed to the background factor that the patients with D1 lymph node dissection were significantly older (p < 0.01) and those who may have more comorbidities. In a comparison of operation type (total or subtotal gastrectomy) in Stage I, the HRs for total gastrectomy were twice as high as the HRs for subtotal gastrectomy during the entire period. This result is also caused by a high number of deaths from other diseases in patients who underwent total gastrectomy [Total: 11.4% (46/402), Subtotal: 4.9% (60/1230)]. Although background factor bias between groups should be kept in mind, the effect of other disease deaths is minor in the early postoperative observation period. It does not sway the conclusion of the optimal intensive follow-up period of this study.

Our study has several limitations that warrant further consideration. First, the retrospective data from a single institution used here may be subject to significant bias, and the results may lack generalizability. Second, our surveillance protocol followed the JGCA guidelines, and data from this study were obtained using a relatively intensive surveillance strategy (which is also a strength of the study). Third and most importantly, our findings could not demonstrate whether postoperative surveillance truly leads to improved survival rates or patient-centered outcomes because it was not intended for that purpose. In colorectal cancer, a 2016 Cochrane analysis found no improvement in overall survival with the implementation of enhanced surveillance of post-curative surgery patients [13]. As for GC, no report has ever proven the effectiveness of intensive follow-up with evidence, and rather, there are scattered reports denying strict follow-up [14, 15]. In the current scenario, when chemotherapy has advanced dramatically with the advent of immune checkpoint inhibitors, early detection of GC relapse through intensive surveillance may lead to a more improved prognosis than ever. Prospective randomized trials comparing intensive surveillance with less intensive surveillance (e.g., testing only when symptomatic) are needed to evaluate the genuine impact of the early detection of occult recurrence on survival and quality of life.

## Conclusions

Our study, using the hazard function, revealed varying trends of HRs for recurrence by GC stage, which can aid in the implementation of scientifically based follow-up after curative gastrectomy for GC.

## Supplementary Information

Below is the link to the electronic supplementary material.

Supplementary file1 (PDF 225 KB)

## Data Availability

The datasets generated during the current study are available from the corresponding author on reasonable request.
