# Supplementary material for: Hazard rates of recurrence for gastric cancer after curative resection: implications for postoperative surveillance
Source: Gastric Cancer. 2024 Dec 26;28(2):275–82. doi: 10.1007/s10120-024-01576-5 (PMC11842406; doi:10.1007/s10120-024-01576-5)

Supplementary Fig.1

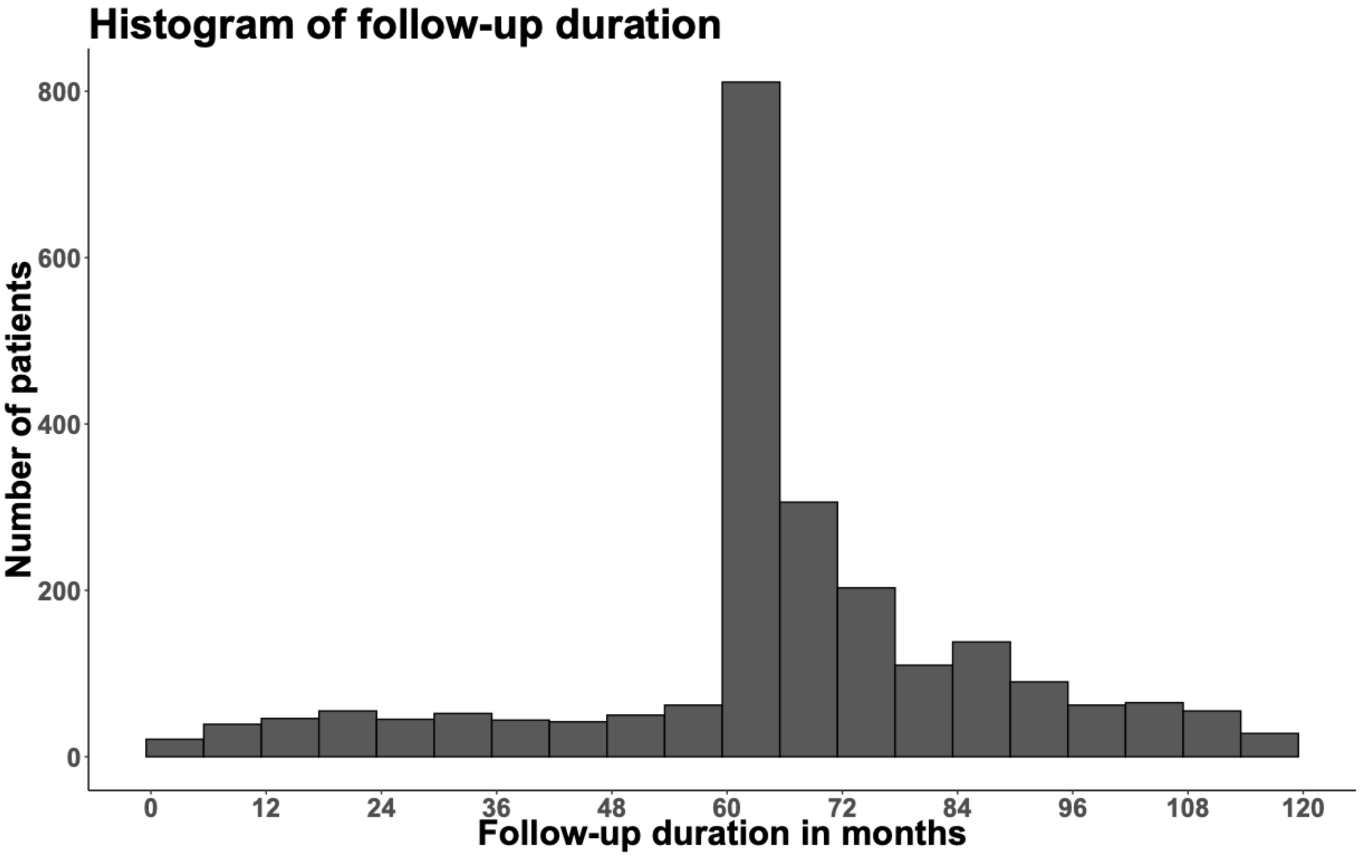

Supplmentary Fig.2

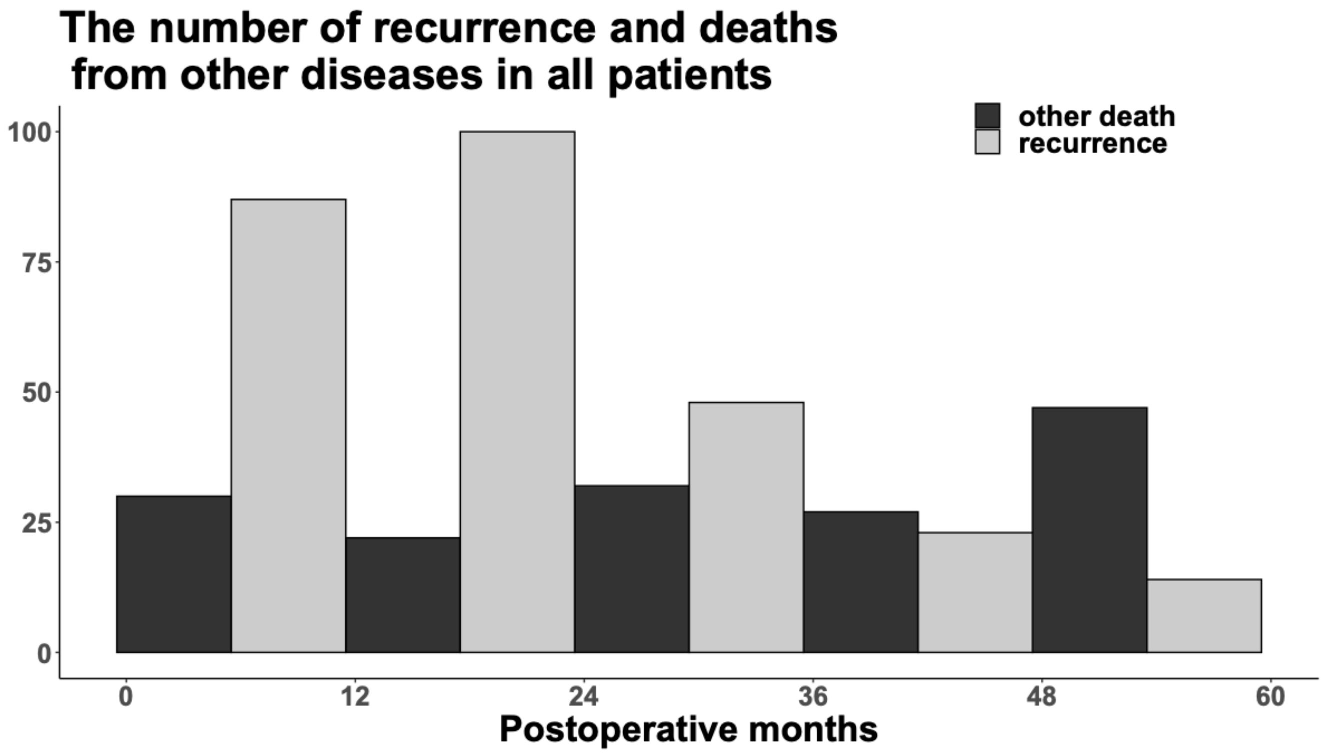

Supplementary Fig.3

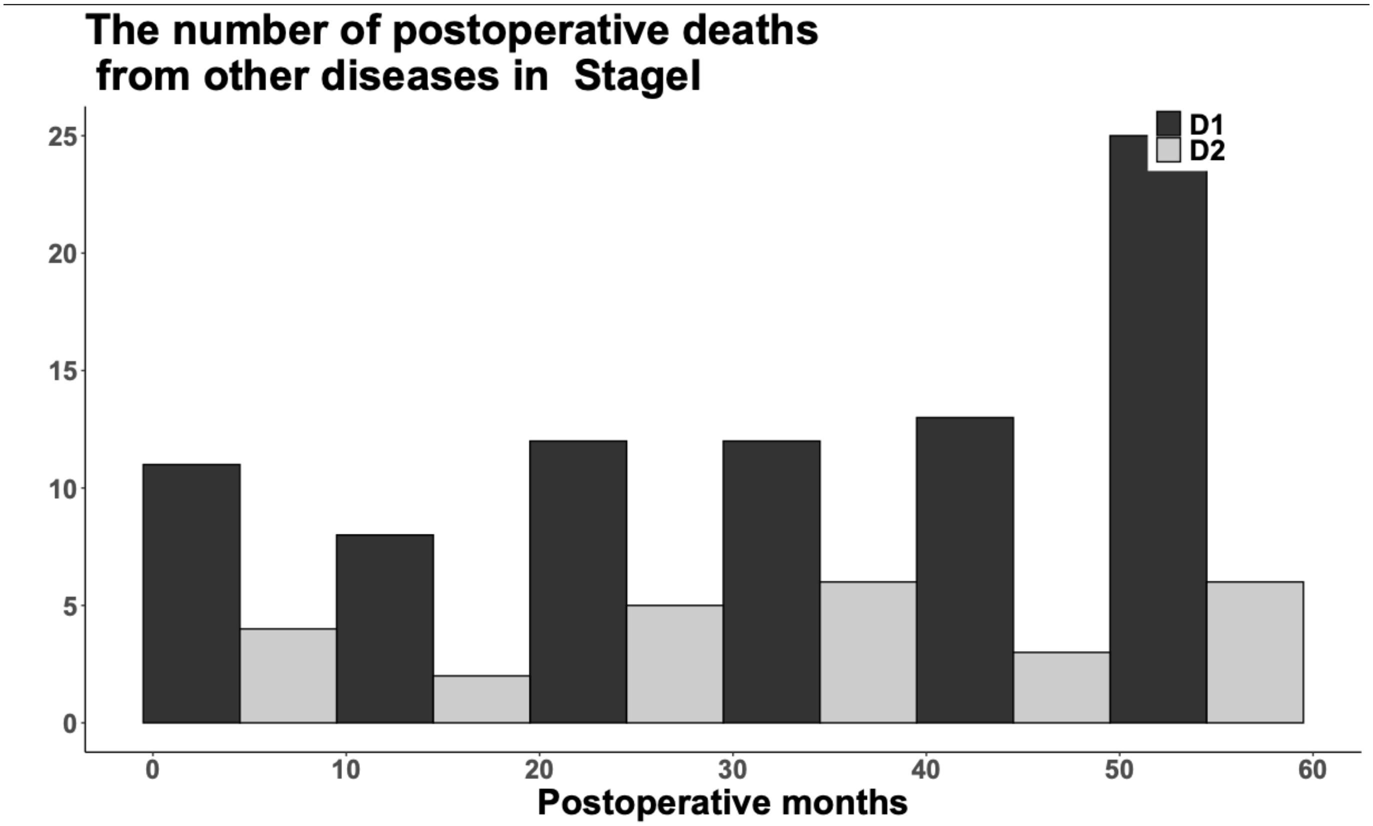

Supplement: Supplementary file 1 — Supplementary file1 (PDF 225 KB) [file 10120_2024_1576_MOESM1_ESM.pdf]
